# Potential for Improving Intrauterine Device (IUD) Service Delivery Quality: Results from a Secondary Data Analysis

**DOI:** 10.12688/gatesopenres.12997.3

**Published:** 2020-03-04

**Authors:** Manish Gehani, Manoj Pal, Anupama Arya, Shobhana Singh, Kaushik S., Kathryn A. O’Connell, Levent Cagatay, Sumon Sengupta, Sunita Singal

**Affiliations:** 1 EngenderHealth Inc., Ahmedabad, Gujrat, India; 2 EngenderHealth Inc., New Delhi, Delhi, India; 3 EngenderHealth Inc., Jaipur, Rajasthan, India; 4 EngenderHealth Inc., Washington, DC, USA; 5 EngenderHealth Inc., Izmir, Turkey

**Keywords:** Quality of IUD services, complications of IUD insertions, secondary data analysis, client follow-ups

## Abstract

**Background: **To accelerate progress toward Family Planning 2020 (FP2020) goals, the government of India focused on improving the quality of intrauterine device (IUD) services. EngenderHealth, an international sexual and reproductive health and rights organization, has been supporting the governments of Gujarat and Rajasthan since 2014 through the Expanding Access to IUD Services in India (EAISI) project by building the capacity of service providers, monitoring compliance with standard practices, and strengthening health systems. This study sought to assess whether EAISI-trained providers offer higher quality IUD services than non-EAISI-trained providers, as indicated by a reduction in confirmed IUD complications.

**Methods: **The study team conducted an analytical cross-sectional study of secondary data collected from follow-up registers at 176 intervention facilities (38 in Gujarat and 138 in Rajasthan) during Phase I of the EAISI project. The analysis included follow-up clients who returned to the same facility between April 2018 and March 2019. We performed a multivariate logistic regression to determine factors associated with IUD complications.

**Results: **During the period of assessment, 56,733 clients received IUD insertions, and 10,747 (18.9%) clients returned for follow-up services. Of the returning clients, 49.4% (N=5,305) had received IUDs from EAISI-trained providers, while 50.6% (N=5,442) had received IUDs from non-EAISI-trained providers. A total of 4.0% (N=432) of all returning clients experienced complications (expulsion: 1.3%, missing strings: 1.7%, infection: 1.1%). Clients who received IUDs from non-EAISI-trained providers were 55.5% more likely (95% CI [26.2%, 91.5%], p<0.0005) to have experienced complications than clients who received insertions from EAISI-trained providers. The type of IUD, the timing of the insertion, and the timing of the follow-up visit also affected complication prevalence.

**Conclusion: **Our findings indicate that intensive, practical clinical skills training for IUD insertion can reduce the prevalence of complications.

## Introduction

India is the second most populous country in the world, contributing to 18% of the global population ^1^. In 1952, India became the first country in the developing world to introduce a national family planning program to lower fertility and stabilize population growth ^2^. India’s Family Planning 2020 (FP2020) goals aim to increase access, choice, and quality of family planning services. Since first making an FP2020 commitment in 2012, India has continued its efforts to expand the range and reach of contraceptive options by introducing new contraceptives and delivering a full range of family planning services at all levels. For example, India has integrated family planning into the Reproductive, Maternal, Newborn, Child, and Adolescent Health (RMNCH+A) Strategy ^3^.

Global evidence has shown that ensuring the availability and quality of intrauterine device (IUD) services can improve family planning uptake, increase modern contraceptive method use, and reduce unmet need for family planning services. Understanding this, as part of its overall commitment to increasing access to and use of family planning services, the government prioritized improving access to quality IUD services. While IUD use among married women in India is low (1.5%), evidence suggests that discontinuation rates for IUDs are lower (26%) than all other contraceptive methods, such as injectables (51%), condoms (47%), and oral contraceptives (42%) ^4^.

Increasing the quality of IUD services can improve client satisfaction, reduce complications, and increase demand for IUD services and continuation of IUD use ^5^. Many factors contribute to the quality of IUD service delivery, including the enabling environment, safeguarding of clients’ rights, and provider skill levels ^6^. Evidence has also suggested that clients who obtained IUDs from untrained providers are significantly more likely to experience IUD complications ^7^ and those complications can lead to unwanted pregnancies ^8^, compromise client safety ^9^, and impose an additional economic burden on clients ^10^. Therefore, reducing IUD complications is important to improving client satisfaction and reducing negative perceptions about IUDs within the community, which in turn can increase demand for IUDs ^9^.

Since 2014, EngenderHealth, an international sexual and reproductive health and rights organization, has been providing technical assistance to the government of India to improve access to quality IUD services. EngenderHealth implemented the Expanding Access to IUD Services in India (EAISI) project in Gujarat and Rajasthan through which EngenderHealth has built the capacity of service providers to deliver quality IUD services, monitored their compliance with standard practices, and strengthened the public health system by training administrators. More specifically, the EAISI project trains public sector service providers in assessing client needs, counseling clients, completing IUD insertions, adhering to infection prevention practices, and ensuring protection of clients’ sexual and reproductive health rights. As part of EAISI’s clinical training, providers practice on anatomic models and clients.

Estimating complications associated with clinical procedures is critical, as this is an established outcome measure of quality of care ^11^. For the EAISI project, the rate of complications served as a proxy measure of effectiveness in improving quality of IUD services. Few (if any) other studies that have investigated the quality of IUD services have explored the importance of provider training and the extent to which such training influences the incidence of IUD complications ^5, 6^. Healthcare providers in India routinely collect follow-up information from IUD clients; the existing availability of such data provided an opportunity for investigating factors associated with IUD complications. The primary aim of this study was to examine factors associated with IUD complications, including the extent to which provider training influenced the rate of IUD complications. A secondary aim of the study was to estimate the magnitude of complications.

## Methods

### Study design

This study was an analytical retrospective cross-sectional study based on secondary data from follow-up visits captured in EAISI’s intervention facilities between April 2018 and March 2019. This manuscript adheres to the Standardized Reporting of Secondary Data Analysis (STROSA) guidelines ^12^.

### Data source

Facilities document data on insertions and follow-up visits in case records and registers. The format of IUD register mirrors national guidelines. This study used the follow-up registers for IUD clients, which documented the follow-up care of the reference population (i.e., IUD clients who returned to EAISI-supported intervention facilities for follow-up services). The register captures the following data: (1) client identification details, (2) details of the insertion, (3) timing of the follow-up visit, and (4) findings from the follow-up visit, including any complications and/or reason of removal, if applicable. The health departments of Gujarat and Rajasthan own the data.

### Approvals, confidentiality, and data availability

India’s Ministry of Health and Family Welfare (MOHFW) provided permission to publish the data. In accordance with the Declaration of Helsinki, since the study used secondary data, an institutional ethics approval was not required. The study team maintained client privacy throughout the analysis; no client-identifiable data were disclosed and the data was anonymized without distorting scientific meaning using the safe harbor method. The study team deposited the dataset into the Harvard Dataverse repository under a CC0 1.0 Universal License ^13^.

### Data flow

EngenderHealth staff collected data from health facilities every month. Data entry operators recorded the data in EAISI’s project database, as mandated by the MOHFW’s Family Planning Department. The study team exported a Microsoft Excel file from this database that comprised data related to follow-up visits that occurred during the relevant period and analyzed the data using SPSS version 24.

### Inclusion and exclusion criteria

The study comprised 176 facilities wherein 1,306 providers trained by EAISI during the first phase of the project (June 2014 to November 2016) worked. This included 38 facilities in Gujarat (14 district hospitals, 5 sub-district hospitals, and 19 community health centers) and 138 facilities in Rajasthan (1 sub-district hospital, 130 community health centers, and 7 primary health centers). The study only included data on clients who returned to the facility for clinical follow-up. Further, the study excluded client data if any of the following criteria were met: a health provider contacted the client for a follow-up phone call, a health provider visited the client for home-based follow-up, or the client visited a different facility (than the one where they received their IUD) for follow-up care. The study team completed a full survey of the available data in the targeted facilities for the study period using universal sampling.

### Analysis unit and variables analyzed

The unit of analysis was a client who, as confirmed through clinical follow-up, experienced a complication following an IUD insertion. The study team defined “complication” for this purpose as the presence of any one or a combination of the following: expulsion, infection, and/or missing strings. This variable (i.e., complication(s)) served as a proxy indicator for the quality of IUD services ^14, 15^. The team the disaggregated client data into two categories for analysis: clients who received IUD insertions from EAISI-trained providers versus clients who received IUD insertions from non-EAISI-trained providers. The latter group included providers who either had not received formal training on IUD insertions or who had received IUD training from a different institution.

The study team also investigated other variables, including the timing of the IUD insertion (postplacental, immediate postpartum, intracesarean, interval), the timing of the follow-up visit after insertion, client age, client state of residence, and the type of IUD inserted (IUD 380A or IUD 375).

### Study size

The number of entries recorded in EAISI’s database for the study period determined the study size.

### Statistical analysis

The study team completed a multivariate logistic regression analysis to determine the probability of complications (reference category: no complication) by training status, after controlling for the following variables: timing of insertion, timing of follow-up after insertion, client age, state, and type of IUD inserted. The team estimated an odds ratio and p value as well as chi square and Nagelkerke R ^2^ values. The team used SPSS 24 to perform the entire statistical analysis.

## Results

### Selection of study population

**Figure 1.**
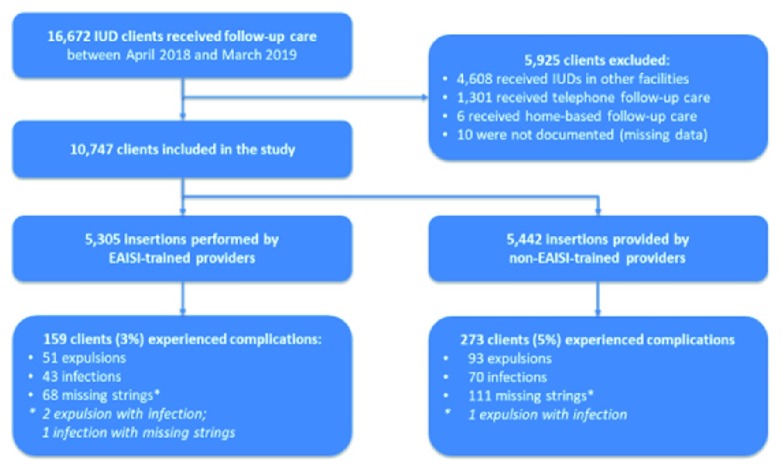
Pathway of Study Participants Selection.

The study team used the inclusion and exclusion criteria previously discussed and detailed in Figure 1 to identify the study population. Of 16,672 clients in the database, 10,747 client records (4,734 from Gujarat and 6,013 from Rajasthan) met the inclusion criteria and were included in the secondary analysis.

### Descriptive results

Ten facilities (four in Gujarat and six in Rajasthan) did not document IUD follow-up cases during the study period due to a lack of follow-up registers and poor documentation practices. The remaining 166 facilities (34 in Gujarat and 132 in Rajasthan) reported that of the 56,733 clients (14,502 in Gujarat and 42,231 in Rajasthan) who received IUDs in these facilities, only 10,747 (18.9%) returned to the facility for clinical follow-up care during the study period. Among these returning clients, 432 (4%) experienced IUD complications: 144 experienced expulsions (1.3%), 113 clients experienced infection (1.1%), and 179 experienced missing strings (1.7%). Table 1 demonstrates the sample distribution, according to the different variables. Raw data are available in the Harvard Dataverse ^13^.

**Table 1.** Distribution of Clients and Proportion of Complications

Variable	Number of clients (% of total) N=10,747	Number of complications/n (% of complications)
**State**		
Gujarat	4734 (44.0%)	222/4734 (4.7%)
Rajasthan	6013 (56.0%)	210/6013 (3.5%)
**Provider training status**		
EAISI-trained	5305 (49.4%)	159/5305 (3%)
Non-EAISI-trained	5442 (50.6%)	273/5442 (5%)
**Timing of insertion**		
Postplacental	7112 (66.2%)	254/7112 (3.6%)
Immediate postpartum	2395 (22.3%)	112/2395 (4.7%)
Intracesarean	498 (4.6%)	44/498 (8.8%)
Interval	693 (6.4%)	19/693 (2.7%)
Unspecified	49 (0.5%)	3/49 (6.1%)
**Type of IUD**		
IUD 375	3454 (32.2%)	113/3454 (3.3%)
IUD 380A	7074 (65.8%)	310/7074 (4.4%)
Unspecified	219 (2.0%)	9/219 (4.1%)
**Timing of follow-up after insertion**		
Within 6 weeks	3142 (29.2%)	147/3142 (4.7%)
6 weeks to 6 months	6440 (59.9%)	267/6440 (4.1%)
More than 6 months	1114 (10.4%)	17/1114 (1.5%)
Unspecified	51 (0.5%)	1/51 (2%)
Type of complication *		**Number of complications (% of** **complications) N=10,747**
Any one complication		432 (4%)
Expulsions		144 (1.3%)
Missing strings		179 (1.7%)
Infections		113 (1.1%)
**Age in years**	**Mean**	**Range**
	24.58	16–45

IUD, intrauterine device * *3 cases had both expulsion and infection, while 1 case had both infection and missing strings*

### Bivariate analyses

Table 1 illustrates a cross-tabulation of variables associated with complications reported following IUD insertion. Clients in Gujarat reported a higher complication rate (4.7%) than those in Rajasthan (3.5%). The study team identified 159 complication cases (3%) among insertions completed by EAISI-trained providers and 273 complications (5%) among insertions completed by non-EAISI-trained providers (those who were either not formally trained or trained by other sources). The most common complication in both groups was missing strings, with 1.3% of EAISI-trained providers’ clients and 2.0% of non-EAISI-trained providers’ clients reporting this complication. Complication incidence varied considerably in relation to the timing of insertion—with the highest complication rates observed among intracaesarian clients (8.8%) followed by immediate postpartum (4.7%), postplacental (3.6%), and then interval clients (2.7%). IUD 380A clients reported a higher rate of complications (4.4%) than IUD 375 clients (3.3%). The frequency of complications also declined according to the timing of the follow-up visit—complication rates were highest among clients receiving follow-up care within six weeks of insertion (4.7%) and lowest among clients receiving follow-up care after six months of insertion (1.5%).

### Multivariate analyses

The study analysis included 10,422 cases of IUD complications (χ ^2^ = 82.996, p <0.0005, Nagelkerke R ^2^ 0.028). Clients who received an IUD from a non-EAISI-trained provider were 55.5% more likely to experience a complication than clients who received an IUD from an EAISI-trained provider. IUD 380A clients were 43.5% more likely than IUD 375 clients to experience a complication. Intracesarean clients were 135.7% more likely than postplacental period clients to experience a complication. The other two categories of insertion timing (immediate postpartum and interval) did not demonstrate a statistically significant association with incidence of complication. Clients who sought follow-up care within six weeks of insertion or between six weeks and six months of insertion were 175% and 178% (respectively) more likely to report a complication than clients who sought care more than six months after insertion. See Table 2 for additional details.

**Table 2.** Multivariate Logistic Regression of IUD Complications.

Variable	Odds ratio	95% CI	p value
**State**			
Gujarat	0.996	0.772, 1.284	0.975
Rajasthan *			
**Provider training status**			
EAISI-trained *			
Non-EAISI-trained	1.555	1.262, 1.915	<0.0005
**Timing of insertion**			
Postplacental *			
Immediate postpartum	1.189	0.909, 1.554	0.206
Intracesarean	2.357	1.607, 3.459	<0.0005
Interval	0.893	0.548, 1.458	0.652
**Type of IUD**			
IUD 375 *			
IUD 380A	1.435	1.143, 1.801	0.002
**Timing of follow-up after insertion**			
More than 6 months *			
6 weeks to 6 months	2.778	1.664, 4.639	<0.0005
Within 6 weeks	2.752	1.623, 4.665	<0.0005
**Age** (continuous variable)	0.974	0.948, 1.002	0.068

* *Reference category*

## Discussion

This study explored the frequency of complications among IUD clients across two states in India. Our findings highlight the frequency of complications among these clients and present factors associated with complications, including the training the provider received, the type of IUD, the timing of insertion, and timing of follow-up care.

Globally, 2% to 8% of IUD clients will experience missing strings within first year of insertion ^16^, 1.6 of every 1,000 IUD clients will experience infection each year ^17^, and 2% and 10% of IUD clients will experience expulsions in their first year ^18^. Missing strings was the most common complication experienced by clients who received IUDs from either EAISI-trained providers and non-EAISI-trained providers. Our data suggest that the occurrence of missing strings, infection, and expulsion among our sample were lower than other studies. This is perhaps reflective of the efforts that the Indian government and EngenderHealth have made to ensure the quality of IUD services.

Our results also indicate that several factors are associated with the frequency of IUD complications. Of note, complications were significantly more common among clients who obtained their IUD from non-EAISI trained providers, a promising outcome from the EAISI project. These positive findings may reflect several efforts by the project to ensure that the providers are delivering high-quality counseling and clinical services to clients. The findings also demonstrate the importance of delivering intensive, practical, clinical training to providers to ensure they are able to deliver quality services thereby reducing subsequent complications.

The finding that complications were significantly more common among women who returned for follow-up care within six weeks may reflect that clients who experienced discomfort or pain or who noted the expulsion returned immediately for follow-up consultation. Other studies have also observed in the finding that complications were significantly more frequent among clients with the IUD 380A ^19^. For example, while a clinical trial, implemented in Nigeria, evaluating the effectiveness of the copper T 380A and multiload copper 375 (MLCU 375) IUD concluded that both types of IUD demonstrated comparable in performances in the first year of use, the trial also identified several issues with the 380A. Clients who received the 380A reported higher discontinuation rates after one year, greater abdominal pain during menstruation and bleeding, and higher expulsion and termination rates than clients who received the 375 IUD ^19^. The results of this study, and those of other studies, suggest that additional clinical research should explore the effectiveness of the copper T 380A in the context of India and other countries.

The timing of insertion was another significant variable associated with complications, with women who received their IUD during the intracesarean period experiencing the highest percentage of complications. This finding is consistent with other research, which reports unacceptably high expulsion and displacement rates among clients who receive IUDs during this period. For example, one study observed a 20% expulsion rate within the 12-week follow-up period among copper IUDs inserted during the intracesarean period ^20^. Another study reported an IUD expulsion rate at one year of 17.6% in intracesarean clients who received the 380A IUD ^21^. As a result, the authors deemed the method as unacceptable for general use. These findings are of concern, given that the expulsion rates after caesarean delivery could prevent proper healing of the laparotomy wound by resulting in a decreased interpregnancy interval, and which can cause significant problems during subsequent pregnancies ^22^.

Client age was not significantly associated with incidence of complications, contradicting other research that observed significantly higher rates of expulsion among women under the age of 18 ^23^. However, few of our sample clients—who overall ranged from 16–45 years of age with an average age of 25—were under the age of 18, which may explain the lack of significant differences associated with age among our sample.

This study had several limitations. First, of the 176 facilities sampled, 10 lacked records of follow-up clients. In addition, 81% of clients who had received insertions from the remaining 166 facilities were not documented due to a variety of reasons, including low client return rates, poor documentation practices, and/or incomplete documentation. Although providers stress the importance of returning to the facility for a follow-up visit after 1.5 months, clearly many clients do not return. This lack of follow-up may be attributable to a number of factors, including economic, geographic, and logistical issues ^24, 25^. As such, our estimation of complications may be biased towards women who were able to return for follow-up care.

Furthermore, we could not comprehensively examine long-term expulsion rates, as the vast majority of clients sought follow-up care within four to six weeks. Due to resource constraints and incomplete secondary data, the team could not ensure a matched sample or conduct a case control study. The study team also recognized that other factors might have caused the complications observed. For example, sexually transmitted infections may have caused infections observed and IUD failures may have led to missing strings (although no such cases were reported). We also acknowledged that these confounders may have been unevenly distributed among the IUD clients who received insertions either from EAISI-trained providers or from non-EAISI-trained providers. The limited fields in the hospital registers hindered our ability to control for these confounding variables. Further, the non-EAISI-trained providers included in this study may have participated in other IUD trainings (for example, during preservice and/or in-service trainings) and received similar information as EAISI-trained providers, thereby misrepresenting the effect of EAISI-provided IUD training on incidence of complications in our analyses. Finally, the study team acknowledges that our analysis framework was constrained as we relied on secondary data and could not ensure the completeness of data.

Despite these limitations, the study demonstrates a cost-effective method of monitoring the quality of IUD services in healthcare facilities and illustrates the effectiveness of IUD services across patient settings. While the other studies examining quality of IUD services have derived data by interviewing providers, observing interactions with clients, or tracking hospital operations indicators, this study employed data from follow-up registers completed after clinical evaluation by the medical experts who assessed and diagnosed the client’s complication(s) or condition. Future studies can adopt this analytical framework to evaluate the outcomes of any skill-based training.

## Conclusion

EAISI’s training, follow-up practice, and monitoring approaches proved to enhance providers’ skills and improve the quality of IUD services. The EAISI model can be introduced in other geographic areas, by the government or other partners, to replicate similar results. Our use of secondary data is a cost-effective method for monitoring the quality of IUD services. These findings establish a foundation for further research and present a feasible model for future assessments of the quality of IUD services in family planning programs.

To improve similar secondary analyses in the future, follow-up registers must be available at all participating facilities, facilities need to improve their documentation practices, and facilities should receive ongoing support to ensure accountability for this record keeping. The data generated should be shared with implementers, policymakers, and other key stakeholders to inform decision making and planning and to enhance the quality of services.

## Data availability

Dataverse: Potential for Improving Intrauterine Device (IUD) Service Delivery Quality: Results from a Secondary Data Analysis, https://doi.org/10.7910/DVN/JYZP7N ^13^.

Data are available under the terms of the Creative Commons Zero “No rights reserved” data waiver (CC0 1.0 Public domain dedication) ^13^.
