# Supplementary material for: Potential for Improving Intrauterine Device (IUD) Service Delivery Quality: Results from a Secondary Data Analysis
Source: Gates Open Res. 2020 Mar 4;3:1473. Originally published 2019 May 24. [Version 3] doi: 10.12688/gatesopenres.12997.3 (PMC6784301; doi:10.12688/gatesopenres.12997.3)
Supplement: Peer review history

# Peer review: Reviewer response for version 3

**DOI:** 10.21956/gatesopenres.14295.r28648

**Published:** 2020-03-19

**Authors:** Avita Rose Johnson

**Affiliations:** 1 Department of Community Health, St. John’s Medical College, Bangalore, Karnataka, India

No more suggestions.

I confirm that I have read this submission and believe that I have an appropriate level of expertise to confirm that it is of an acceptable scientific standard.

# Peer review: Reviewer response for version 2

**DOI:** 10.21956/gatesopenres.14203.r27868

**Published:** 2019-10-04

**Authors:** Nuriye Ortayli

**Affiliations:** 1 Freelance Consultant, Instabul, Turkey

Authors have addressed the issues they could. Changing the study design is a huge task and I understand that they cannot embark on it at the time being, but I hope they will try it in the future given the availability of huge dataset. I think it can be indexed.

I confirm that I have read this submission and believe that I have an appropriate level of expertise to confirm that it is of an acceptable scientific standard.

# Peer review: Reviewer response for version 2

**DOI:** 10.21956/gatesopenres.14203.r27869

**Published:** 2019-10-01

**Authors:** Avita Rose Johnson

**Affiliations:** 1 Department of Community Health, St. John’s Medical College, Bangalore, Karnataka, India

Making changes as suggested, has resulted in a new and improved version of the original paper.

I confirm that I have read this submission and believe that I have an appropriate level of expertise to confirm that it is of an acceptable scientific standard.

# Peer review: Reviewer response for version 1

**DOI:** 10.21956/gatesopenres.14105.r27425

**Published:** 2019-07-22

**Authors:** Nuriye Ortayli

**Affiliations:** 1 Freelance Consultant, Instabul, Turkey

I think this is a study addressing an important issue. For decades substantial resources have been committed to training of providers in method provision and specifically in provision of IUDs with little evidence regarding its impact. 

 Working with a large database to measure the effect of provider training is going to be a meaningful contribution to the literature.

 However I have a few questions. First one is the most important and it is related to study design:

 Based on the research question (what is the effect of provider training on complications of IUD) and the database available, in my view study design should have been case control. Thus instead of assessing the contribution of several factors on IUD complications (such as provider training, age of user, time of insertion etc), researchers could have compared cases (those with complications) with controls (a matched sample of those without complications). As authors say that percentage of IUDs provided by trained and non-trained providers are almost equal and therefore, given that a higher percentage of complicated cases received services from non-trained providers we can assume that training of a provider is an important factor. However, I think a case control design could have given us a result without making that assumption. 

 Therefore, I believe this paper can benefit a lot from a review by a statistician/epidemiologist regarding study design. 

 Other points I want to raise are:

1. Why pregnancy with IUD in situ is not considered among the complications. If there were no such cases it should be stated so. 
2. Being an IUD provider for thousands of women, and a trainer of many years, I find it hard to believe that IUD strings can disappear due to manipulation by the woman, or infection can be the result of menstrual hygiene and/or sexual practices (if the authors want to say STIs, then they should state it clearly)

Beyond these points I think this is an important issue, and researchers have pulled together a database of impressive size. With advise from peer epidemiologists, I think results can be more convincing for the audiences.

I confirm that I have read this submission and believe that I have an appropriate level of expertise to confirm that it is of an acceptable scientific standard, however I have significant reservations, as outlined above.

# Response

**Published:** 2019-08-30

**Authors:** Manish Gehani

**Affiliations:** EngenderHealth Inc, India

Dear Nuriye

Thank you for the comments. The authors fully appreciate that a case-control study would have strengthened the conclusions that could be made, and this limitation will be added to the paper. Given several resource constraints, we were unable to implement a study using a case-control methodology. Rather, we took a cost-effective and opportunistic approach to utilize valuable, secondary data. Several steps were taken during our analysis to address any potential confounding factors, such as age, timing of insertion, type of IUCD. We believe that the application of this analysis helps to alleviate the potential confounded effects related to the incidence of complications as a result of provider training. The current study design is robust as per epidemiology and research methodology.

IUD failure was not the complication taken into consideration in the study as a proxy indicator and there was no such case reported in the study.

We will correct the text regarding the other potential causes of infection and missing strings, besides as a complication of IUD insertion, as this was a mandatory reporting item as per STROSA guidelines.

Regards

On behalf of all authors-

Dr Manish Gehani

# Peer review: Reviewer response for version 1

**DOI:** 10.21956/gatesopenres.14105.r27248

**Published:** 2019-05-28

**Authors:** Avita Rose Johnson

**Affiliations:** 1 Department of Community Health, St. John’s Medical College, Bangalore, Karnataka, India

Summary:

 This study looks at whether women who had IUD inserted by EAISI trained health care providers, had lower complications than women whose IUD was inserted by a non-EAISI trained provider. The rates of complication being a surrogate indicator for the quality of IUD services provided. An analysis of secondary data from follow-up registers of 176 health facilities, revealed 10,747 client follow-ups, half of the IUD insertions were conducted by EAISI trained providers and half by non-EAISI trained providers. Complications among the latter were significantly higher. This study indicates the need for hands-on training for health providers to improve the quality of IUD services.

 Suggestions:

1. Need to state specifically if HMSC (Health Ministry Scientific Committee) permission was obtained, as the data has been shared and stored overseas.
2. Table 1 and 2 can be combined by adding a total N(%) column in table 2.

I confirm that I have read this submission and believe that I have an appropriate level of expertise to confirm that it is of an acceptable scientific standard.

# Response

**Published:** 2019-05-30

**Authors:** Manish Gehani

**Affiliations:** EngenderHealth Inc, India

Dear Dr Avita

We informed Health Ministry prior to using the data for research and sought the due approvals. This has been mentioned in the section on Approvals, confidentiality, and data availability as per STROSA guidelines.

We will try to club both the tables as suggested.

Many thanks and best regards
